# Stop Sexual Harassment: A study protocol for a cluster randomised controlled trial in secondary schools in Norway

**DOI:** 10.3389/fpubh.2022.1051983

**Published:** 2023-01-09

**Authors:** Hilde Slaatten, Bente Storm Mowatt Haugland, Ragnhild Bjørknes, Tonje Fyhn, Torill Helene Tveito, V. Paul Poteat, Kyrre Breivik

**Affiliations:** ^1^Regional Centre for Child and Youth Mental Health and Child Welfare, NORCE Norwegian Research Centre, Bergen, Norway; ^2^Department of Clinical Psychology, University of Bergen, Bergen, Norway; ^3^Norwegian Centre for Child Behavioral Development, Oslo, Norway; ^4^Worklife and Inclusion, NORCE Norwegian Research Centre, Bergen, Norway; ^5^Department of Health, Social and Welfare Studies, University of South-Eastern Norway, Horten, Norway; ^6^Department of Counseling, Developmental and Educational Psychology, Boston College, Newton, MA, United States

**Keywords:** sexual harassment, gendered harassment, homophobic harassment, prevention, youth, RCT, Stop Sexual Harassment, intervention

## Abstract

**Background:**

Sexual- and gendered harassment are normalised in many peer groups, yet their associations with mental health concerns among adolescents are well-established. School based interventions that prevent and reduce sexual and gendered harassment among younger adolescents are scarce. For schools, protecting pupils from harassment may be challenging if the behaviour is trivialised among the pupils themselves. In the current study, the school intervention “Stop Sexual Harassment” was therefore developed to help teachers and pupils detect, address and stop sexual and gendered harassment among pupils ages 13–15 in Norwegian secondary schools.

**Methods:**

In this study the effectiveness of “Stop Sexual Harassment” is evaluated *via* a cluster randomised controlled trial among pupils and teachers at 38 secondary schools. Schools were randomised into intervention and control groups. Primary outcomes are sexual and gendered harassment victimisation and perpetration, which will be assessed by the administration of questionnaires to pupils and teachers at baseline, and 2-, and 7-months follow-up. A process evaluation of the intervention implementation will be conducted through focus group interviews with pupils and teachers to gain insight about their experiences with the program components and implementation of the intervention.

**Discussion:**

If the intervention yields positive effects, large-scale implementation of the program may be offered for secondary schools. The program may thus reduce sexual and gendered harassment among young adolescents.

**Clinical trial registration:**

clinicaltrial.gov; identifier: NCT04716400.

## 1. Introduction

In the Nordic countries, sexual and gendered harassment are relatively common among lower secondary school pupils (12–15 years of age) (1–3). This is particularly the case for sexual minority pupils (e.g., lesbian, gay, bisexual, queer, etc.) (4–6). In this study, sexual harassment is defined as unwanted sexual attention that may be physical, verbal and non-verbal. Gendered harassment is defined as harassment based on gender identity, gender expression and sexual orientation. The associations between exposure to sexual and gendered harassment and mental health problems among adolescents are well-established. Longitudinal studies support that sexual and gendered harassment lead to depressive symptoms (4–6) as well as emotional distress (5, 7). Furthermore, being exposed to sexual or gendered harassment is associated with higher levels of suicidal feelings among young people (8, 9). In Norway, schools are obliged by law to protect pupils from being exposed to harassment (10), including sexual and gendered harassment (11, 12). Even though sexual and gendered harassment is significant concerns to adolescents' mental health, and is illegal by Norwegian law, there are few resources or programs available to schools to help prevent or intervene against it (13).

While there has been an increasing focus on how to reduce and prevent dating violence (violence or threats of violence within a dating relationship which may be physical, emotional or sexual) and sexual assaults (intentional sexual contact without consent) in the last decade (14–17), only a few interventions address sexual harassment among secondary school pupils under 16 years of age. The school-based interventions “Dating Matters,” “Green Dot” and to some extent “Shifting Boundaries” have been found to reduce sexual harassment in this age group (18–20). However, a major focus of these programs is to reduce sexual harassment or violence within dating relationships. While some pupils perceive sexual harassment as a part of “natural” heterosexual dating (21–24), sexual harassment also occurs outside of a dating relationship and is normalised among some adolescents (22, 25–28). Intervention programs should thus put a greater focus on sexual harassment among non-dating peers in schools. Also, as some pupils seem to perceive sexual harassment as playful and harmless (22, 27, 29) there is a need for interventions which help pupils identify “normalised” unwanted sexual attention as sexual harassment.

There is limited knowledge concerning what works in preventing gendered harassment. The intervention “Green Dot,” which has been found to reduce sexual harassment perpetration and victimisation (18), only seems to reduce harassment among sexual majority youth, but not among sexual *minority* youth (26). Even though there are several toolkits and campaigns designed to address harassment based on gender, gender expression or sexual orientation, few have been systematically evaluated for effectiveness and reported in the peer-reviewed literature. Of those interventions that have been evaluated, their effectiveness remains uncertain because most evaluations have lacked an experimental design with a control group (30). Thus, there is a need for more knowledge concerning what works in preventing gendered harassment in the school setting.

### 1.1. Aims and hypotheses

The aim of the current cluster Randomised Controlled Trial (RCT) is to evaluate and test the effectiveness of the intervention “Stop Sexual Harassment” in Norwegian schools. The following hypotheses are posed:

- Schools participating in “Stop Sexual Harassment” will reduce sexual and gendered harassment victimisation and perpetration among pupils compared to schools in a wait-list control condition.
- Schools participating in “Stop Sexual Harassment” will increase pupils' prosocial bystander behaviour when a co-pupil is exposed to sexual and gendered harassment compared to schools in the wait-list control condition.
- Schools participating in “Stop Sexual Harassment” will increase teachers' awareness of, and interventions towards sexual and gendered harassment among pupils compared to teachers in the wait-list control condition.
- Schools participating in “Stop Sexual Harassment” will increase pupils' attitudes, norms, behavioural control, and willingness to intervene when a co-pupil is exposed to sexual and gendered harassment compared to schools in the wait-list conditions.

Process evaluation of the intervention implementation will be conducted to provide insight into the delivery, quality, and engagement with program components, in order to understand facilitators and barriers to the successful implementation of the intervention and indicate some explanations regarding *why* the intervention does or does not have an effect. This will provide useful data to make further adaption to the intervention for acceptability and effectiveness if the intervention is to be scaled up (31).

## 2. Methods and analysis: Randomised controlled trial

### 2.1. Intervention

“Stop Sexual Harassment” aims to prevent sexual and gendered harassment among pupils in secondary school, which in Norway involves pupils 13–15 years of age in 8–10^th^ grade. The intervention is delivered as eight structured lessons for pupils, administered by their class teacher at school, and during school hours. The training for teachers and other staff includes participation in a digital course comprising the same topics as the pupil lessons. For information about the steps, time frame and duration of the delivery of “Stop Sexual Harassment,” see Table 1. Each structured lesson consists of an illustrated teacher-led digital lecture that come with individual-, group- and class- assignments and discussions. Each lesson is described thoroughly in manuals for teachers on how to lead and conduct the assignments and discussions.

**Table 1:** Delivery of “Stop Sexual Harassment.”

	**First month of a new school year**	**First 4 month of a new school year**	**From the start of the school year and onging**
Class teachers	Individual participation in a digital course comprising eight lectures and preparation of eight pupil lectures with the same topic. This can be done in 1 day, or over several days during the course of August (Duration: 7 h pluss time for practical preparations prior to each lecture).	Delivery of eight structured lessons to pupils in class. (Duration: 8 lessons X 60 min).	Teachers intervene when sexual and gendered harassment is detected.
Other school staff (including school administration, teachers and assistants)	Individual participation in a digital Course comprising four lectures (Duration: 2–3 h).		Staff intervene when sexual and gendered harassment is detected.
Pupils		Participation in eight structured lessons during school hours (Duration: 8 lessons X 60 min).	Pupils tell a teacher when observing or being exposed to sexual and gendered harassment. When safe; pupils show disapproval when a co-student exposes another pupil to sexual and gendered harassment, or when they are exposed to this themselves.

The program is tailored to help teachers and pupils *detect* gendered and sexual harassment, as well as to train them to *address and stop* the harassment. The eight lectures follow Latane and Darley's classical five-step model that leads to intervention in an emergency (32). See Table 2 for an overview of the content, aims and assumed change mechanisms for the intervention. Applying Latane and Darley's (32) model to the prevention of gendered and sexual harassment, the pupils need to (1) Detect unwanted and harmful behaviours that constitute gendered and sexual harassment, (2) Realise that the harassment needs to stop, (3) Be prepared to tell their teachers about the harassment, (who in turn intervene against the harassment), (4) Obtain *skills* regarding how to signal disapproval when someone (a co-student, or they themselves) are harassed, and finally (5) When safe, signal disapproval when witnessing or being exposed to sexual and gendered harassment. The assumed change mechanisms of the program theory are drawn from the Theory of Planned Behaviour. This theory states that an individual's behaviour can be predicted by their behavioural intentions. These intentions are determined by the following three factors: (a) attitudes towards the behaviour (e.g., the extent to which an individual approves or disapproves of the behaviour); (b) subjective norms (e.g., the extent to which an individual perceives others to approve or disapprove of the behaviour or how to respond to it); and (c) perceived behavioural control (e.g., the extent to which an individual have the skills to or feels capable of acting) (33). In line with this theory, the program aims to influence attitudes, norms and perceived behavioural control when it comes to signaling disapproval and/or telling a teacher when a co-pupil harasses another pupil, or if pupils are exposed themselves. The program theory of “Stop Sexual Harassment” also builds on bullying prevention research (34–37) and bystander psychology (38–40).

**Table 2:** The content, aims, and assumed change mechanism for “Stop Sexual Harassment” intervention.

**Latane and Darley (32) five step model that leads to intervention in an emergency**	**Lessons**	**Aim of class activities**	**Assumed change mechanism**
	Lesson 1: Prevention of sexual and gendered harassment.	To create a safe space for future lectures	
Step 1: Noticing the event.	Lesson 2: Detecting sexual harassment.	To become aware that sexualized attention is unwanted for many pupils.	KNOWLEDGE: More pupils will recognise sexual harassment behaviour. NORMS: More pupils will challenge the view that sexual harassment is “normal adolescent behavior.”
Step 1: Noticing the event.	Lesson 3: Detecting gendered harassment.	To become aware how gendered harassment is manifested and how it harms sexual minorities, and anyone who violates traditional gender normative behaviour.	KNOWLEDGE: More pupils will recgonize gendered harassment behaviour. NORMS: More pupils will challenge the view that gendered harassment is “normal adolescent behaviour.”
Step 2: Interpreting the event as an emergency that requires help.	Lesson 4: Sexual and gendered harassment in our school.	To identify behaviour characterised as sexual and gendered harassment in their school which needs to stop.	ATTITUDES: More pupils will perceive harassment behaviour as problematic, which calls for action.
Step 3: Accepting responsibility for intervening.	Lesson 5: The teachers' responsibility.	To practise telling a teacher when a co-student is exposed to harassment.	ATTITUDES: More pupils will believe it is ok to tell their teacher if a co-pupil or they themselves have been exposed to harassment.
	Lesson 6: Knowing what is illegal	To explore what behaviour is characterise as sexual assaults by Norwegian law.	
Step 4: Knowing how to intervene or providing help.	Lesson 7: Our responsibility as a co-student.	To practise how to signal disapproval when a co-pupil harasses another pupil.	ATTITUDES: More pupils will believe it is ok to signal disproval when a co-student harasses another pupil. NORMS: More pupils will believe their co-pupils will support them if they signal disapproval when a co-pupil harasses another pupil. CONTROL: More pupils will have the skills to signal disproval when a co-student is exposed to harassment.
Step 4: Knowing how to intervene or providing help.	Lesson 8: What we can do ourselves.	To be able to use the disapproval statements for future harassment victimization.	CONTROL: More pupils will have the skills to signal disproval when they are exposed to sexual harassment themselves.
Stop 5: Intervene in the situation	After completing all lectures.	To signal disapproval or tell a teacher when a co-pupil or the pupils themselves are exposed to harassment.	INTENTIONS:- More pupils will intend to signal disproval when a co-student harasses another pupil or if they are harassed themselves. - More pupils will intend to tell their teacher if a co-pupil or they themselves are exposed to harassment. BEHAVIOUR: - Fewer pupils will engage in harassment perpetration. -Fewer pupils will experience harassment victimisation. - More pupils will tell their teacher if a co-pupil or they themselves are exposed to harassment. - More pupils will signal disapproval when a co-student harasses another pupil, or if they are harassed themselves.

### 2.2. Study design

This study uses a cluster randomised controlled superiority trial with two parallel groups of schools; one intervention group and one delayed access wait-list control group. The schools in the wait list condition will receive the intervention after the data are collected. The study will thus utilise quantitative data to assess the effect of the intervention.

### 2.3. Sample size and power

Multilevel modelling will be used to analyze the effect of the intervention on sexual and gendered harassment victimisation and perpetration. By taking this approach, we will take into account the hierarchical design of the data due to cluster randomisation by school and school class. In Norway, secondary school pupils are in the same class, with the same peers all week except for a few hours. We approximated cluster effects (Intra Class Correlation; ICC) that may influence the main outcomes (sexual and gendered harassment and emotional problems) by taking advantage of results from previous studies. Previous studies indicate that the school and/or school class level contribute to relatively little (<5%) of the total variance of sexual harassment victimisation, bullying and emotional concerns. However, some studies suggest that this might not be the case for attitudes towards sexual minorities, where between 12.5 and 30% of the total variance has been explained by school/ class levels. Power calculations, applying the Optimal Design Software, show that 38 schools are needed to detect effect sizes of 0.30 (small to medium effects), according to Cohen (41). This is based on a 0.05 level of significance, power = 0.80, ICC level 2 = 0.25, IC level 3 = 0.05, number of cluster level 2 = 6 (classes), and a cluster size=20 (pupils). The statistical power of the analyses can be increased by including the pre-intervention measure of the outcome variables as a covariate. Assuming that the average school level of the outcome variable at pre-intervention explains 50 or 30 percent of the variation of the outcome variable at *post-test*, this decreases the number of schools to 30 and 32, respectively, given the same assumptions as above. We therefore decided to include at least 32 schools (16 intervention schools) to guard against the possibility of Type II errors. Thus, a minimum of 3,840 pupils (32 schools × 120 students) should take part in the study to have sufficient statistical power.

### 2.4. Recruitment

**Figure 1:**
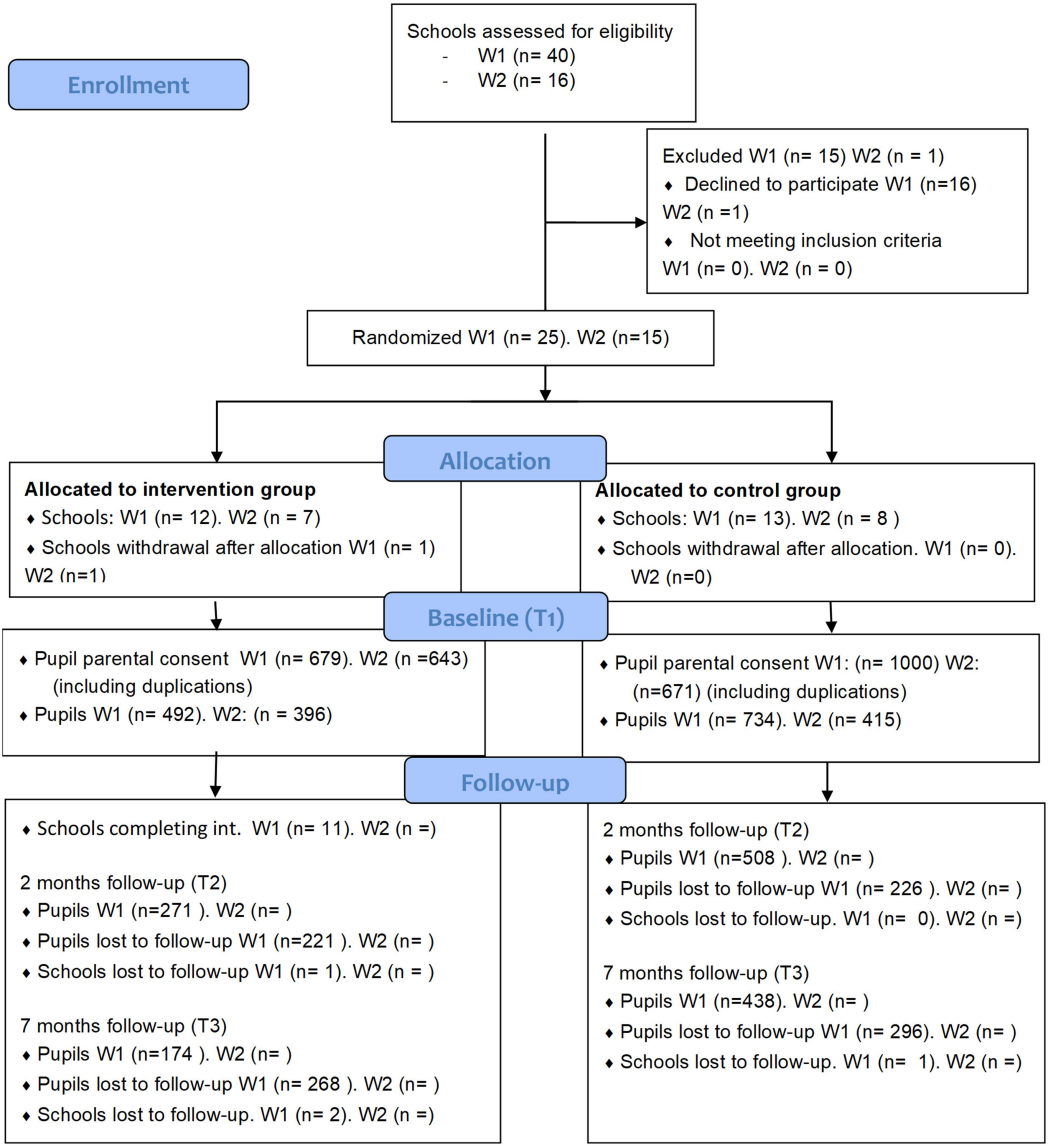
Flow diagram for study participants.

Schools were recruited to take part in the “Stop Sexual Harassment” intervention and the randomised control trial (RCT) at two time points. The recruitment of schools started in March 2019 and ended in May 2022. In the first recruitment phase, schools in the counties of Rogaland, Vestland, Nordland and Buskerud/Viken were invited to participate during autumn 2021. In the second recruitment phase, more schools in these counties, in addition to schools in the cities of Oslo and Trondheim were invited to participate during autumn 2022. Two recruitment phases were needed because several schools in wave 1 withdrew from the study due to the schools' management of the COVID-19 pandemic and its regulations. Please see Figure 1 for a flow chart of the participation rate. In the end, all secondary schools in Norway were made eligible for participation. Schools were invited by postal mail and/or e-mail and follow-up phone calls or by regional and national news channels/newspapers.

Inclusion criteria were public and private upper secondary schools in Norway. Exclusion criteria were schools where the curriculum is taught in languages other than Norwegian, and schools for pupils with disabilities or other health impairments (e.g., hospital schools). In Norway, upper secondary school consist of grade 8–10. All school staff within the intervention schools are encouraged to complete the digital training for “Stop Sexual Harassment” and to deliver the structured lessons to their pupils, regardless of whether or not they agree to take part in the RCT study (i.e., by participating in data collection through completing a survey). Pupils within the participating classes are not granted the opportunity to opt out of participating in the lessons, even if they do not consent to participate in the RCT. As such, all pupils will be exposed to the materials, as the intervention is delivered to the class as a whole, but youth who do not consent to participate in the RCT will not complete any surveys.

All teachers and pupils in 8th and 9th grade (during the time of recruitment) at the participating schools are invited to take part in the RCT. Participation involves completing three questionnaires at three time points. At each school, pupil questionnaires are administered by class teachers who follow written instructions from the principal investigator. Prior to participation, the child's class teachers provide parents with consent forms including information about the study. Parents, pupils, and teachers are informed in their respective consent form that it is voluntary to participate and that they have the right to withdraw from the study at any point in time. This information is repeated orally for pupils prior to completing the questionnaires during school hours. Pupils who do not want to participate in the study are given other assignments prepared by their class teachers during the time allocated for completing the questionnaires.

### 2.5. Randomisation procedure

The intervention “Stop Sexual Harassment” is a whole school approach that involves all members of the school community. Comparisons must thus be at the school and not the individual level and schools were chosen as the cluster of randomisation. Schools were randomised into intervention and control schools by an independent professional [i.e., external statistician working at Services for sensitive data (TSD)]. The randomisation was stratified based on number of participating classes within each school with a 1:1 allocation. All schools were paired based on similarity in size of participating classes and whether they were public or private schools. Syntax (in stata) was used to randomly assign one school in each pair to the intervention group for both wave 1 and wave 2. The remaining school of each pair was assigned to the wait-list control group. Schools were informed about their allocation after completing baseline questionnaires.

### 2.6. Baseline and follow-up assessments

Pupils and teachers will complete separate baseline questionnaires, and two follow-up questionnaires spaced at 2- and 7- months post-intervention. By comparing baseline and follow-up questionnaires between the intervention schools and the control school, we will be able to assess whether the intervention “Stop sexual harassment” is successful in reducing sexual and gendered harassment victimisation and perpetration among pupils. See Table 3 for a timeline of the intervention and the assessments, and Figure 1 for a flowchart of the participation rate. The primary outcomes variable consists of instruments that measure sexual harassment (28, 42) and harassment due to gender non-conformity, sexual harassment and homophobic behaviour (43, 44). For a description of primary, secondary and mediator variables, see Tables 4–6.

**Table 3:** Timeline of the intervention and the assessments.

	**Wave 1**	**Wave 2**
Baseline Assessment (T1)	May–June 2021	May–June 2022
Randomisation	June 2021	June 2022
Training of teachers	August 2021	August 2022
Implementation of “Stop Sexual Harassment”	August 2021–November 2021	August 2022–November 2022
Focus group interviews		December 2022
Short term follow-up (T2)	January- March 2021 (Delay due to the COVID-19 Pandemic)	January 2023
Long term follow-up (T3)	June 2022	June 2023

**Table 4:** Primary outcome variables.

	**Measure**	**Source of measure**	**Data source**
Sexual harassment *victimisation*	The AAUW sexual harassment survey (13 items)	AAUW (42); Hill and Kearl (28)	Pupils
Harassment *victimisation* due to gender non-conformity	Modified version of the Harassment perceived as occurring due to gender non-conformity (4 items)	Based on Martin-Storey and August (43)	Pupils
Harassment *victimisation* due to sexual orientation	Modified version of the Harassment perceived as occurring due to sexual minority status (4 items)	Based on Martin-Storey and August (43)	Pupils
Sexual harassment *perpetration*	The AAUW sexual harassment survey (13 items)	AAUW (42); Hill and Kearl (28)	Pupils
Harassment *perpetration* due to gender non-conformity	Modified version of the Harassment perceived as occurring due to gender non-conformity (4 items)	Based on Martin-Storey and August (43)	Pupils
Harassment *perpetration* due to sexual orientation	Modified version of the Harassment perceived as occurring due to sexual minority status (4 items)	Based on Martin-Storey and August (43)	Pupils
Homophobic behaviour	Homophobic behaviour (5 items)	Paul Poteat et al. (44)	Pupils
Sexual and gendered harassment perpetration (GASH)	Self-constructed global scale measuring: verbal sexual harassment, physical sexual harassment, sexual rumour, homophobic harassment, harassment due to gender non-conformity (6 items)		Pupils

**Table 5:** Secondary outcome variables.

	**Measure**	**Source of measure**	**Data source**
Depressive symptoms	Short Moods and Feelings Questionnaire (13 items)	Angold et al. (45); Larson et al. (46); Lundervold et al. (47).	Pupils
Anxiety symptoms	Screen for Child Anxiety Related Emotional Disorders- short form (5 items)	Birmaher et al. (48); Birmaher et al. (49).	Pupils
Reactions to pupils being exposed to sexual harassment	Reactions questions based on the AAUW sexual harassment survey (26 items)	Based on AAUW (42); Hill and Kearl (28)	Teachers
Reactions to pupils being exposed to harassment due to gender non-conformity	Reactions questions based on Harassment perceived as occurring due to gender non-conformity (8 items)	Based on Martin-Storey and August (43)	Teachers
Reactions to pupils being exposed to harassment due to sexual orientation	Reactions questions based on Harassment perceived as occurring due to sexual minority status (8 items)	Based on Martin-Storey and August (43)	Teachers
Reactions to pupils being exposed homophobic harassment	Reactions questions based on Homophobic behaviour (10 items)	Based on Paul Poteat et al. (44)	Teachers
Reactions to GASH perpetration among pupils	Theory of planned behaviour scale measuring teachers reactions towards sexual and gendered harassment perpetration among pupils (10 items)	Based on Fishbein and Ajzen, 2010 (50)	Teachers
Attitudes towards reacting to GASH perpetration among pupils	Theory of planned behaviour scale measuring attitude towards reacting to GASH perpetration among pupils (6 items)	Based on Fishbein and Ajzen, 2010 (50)	Teachers
Intention to react to GASH perpetration among pupils	Theory of planned behaviour scale measuring intention to react to GASH perpetration among pupils (6 items)	Based on Fishbein and Ajzen (50)	Teachers
Subjective norms regarding reacting to GASH perpetration among pupils	Theory of planned behaviour scale measuring subjective norms regarding reacting to GASH perpetration among pupils (6 items)	Based on Fishbein and Ajzen, 2010 (50)	Teachers
Perceived behavioural control regarding reacting to GASH perpetration among pupils	Theory of planned behaviour scale measuring behavioural control regarding reacting to GASH perpetration among pupils (6 items)	Based on Fishbein and Ajzen (50)	Teachers

**Table 6:** Mediator variables.

	**Measure**	**Source of measure**	**Data source**
Attitudes towards gendered and sexual harassment (GASH)	Theory of planned behaviour scale measuring attitudes towards GASH (6 questions)	Based on Fishbein and Ajzen (50)	Pupils
Reporting GASH to teachers	Theory of planned behaviour scale measuring having told a teacher when a co-student was exposed to GASH (6 questions)	Based on Fishbein and Ajzen (50)	Pupils
Intention to report GASH to teachers	Theory of planned behaviour scale measuring intention to tell a teacher if a co-student is exposed to GASH (6 questions)	Based on Fishbein and Ajzen (50)	Pupils
Bystander GASH behaviour	Theory of planned behaviour scale measuring prosocial bystander behaviour when a co student was exposed to GASH (6 questions)	Based on Fishbein and Ajzen (50)	Pupils
Intention to perform bystander GASH behaviour	Theory of planned behaviour scale measuring intention to perform prosocial bystander behaviour if a co-student is to be exposed to GASH (6 questions)	Based on Fishbein and Ajzen (50)	Pupils
Attitudes towards performing bystander GASH behaviour	Theory of planned behaviour scale measuring attitudes towards performing prosocial bystander behaviour if a co-student is exposed to GASH (6 questions)	Based on Fishbein and Ajzen (50)	Pupils
Subjective norms regarding performing bystander GASH behaviour	Theory of planned behaviour scale measuring subjective norms regarding performing prosocial bystander behaviour if a co-student is exposed to GASH (6 questions)	Based on Fishbein and Ajzen (50)	Pupils
Perceived behaviour control regarding performing bystander GASH behaviour	Theory of planned behaviour scale measuring perceived behaviour control regarding performing prosocial bystander behaviour if a co-student is exposed to GASH (6 questions)	Based on Fishbein and Ajzen (50)	Pupils
Reactions to sexual harassment victimisation	Reactions questions based on AAUW sexual harassment survey (26 items)	Based on AAUW (42); Hill and Kearl (28)	Pupils
Reactions to harassment victimisation due to gender nonconformity	Reactions questions based on the Harassment perceived as occurring due to gender nonconformity (8 items)	Based on Martin-Storey and August (43)	Pupils
Reactions to harassment victimisation due to sexual orientation	Reactions questions based on Harassment perceived as occurring due to sexual minority status (8 items)	Based on Martin-Storey and August (43)	Pupils
Bullying victimisation and perpetration	Bullying victimisation, and perpetration, with and without sexual content (4 item)	Solberg and Olweus (51); Olweus et al. (36)	Pupils
The Bergen Questionnaire on Antisocial Behaviour	(9 items)	Based on Bendixen and Olweus, 1999 (52); Bendixen et al. (53)	Pupils

### 2.7. Data collection procedures

Questionnaires to pupils and teachers are administered electronically, distributed by a survey tool (Nettskjema; https://nettskjema.no/?lang=en). For pupils, data will be collected during school hours. Pupils who are quarantined or home-schooled during the COVID-19 restrictions, or not present at school for other reasons, when the questionnaires are administered, may complete these at home. It takes about 30 min to complete the pupil questionnaire and about 20 min to complete the teacher questionnaire.

### 2.8. Data analysis

Descriptive statistical analyses and frequency tables will be used for comparisons between intervention and control group. Multilevel modelling will be used to assess the impact of the intervention on the primary and secondary outcome variables. Potential subgroup differences (e.g., gender) will be analysed by including interaction effects. The psychometric qualities of the outcome variables will be assessed by descriptive statistics (e.g., floor/ceiling effects), correlation analyses and exploratory and confirmatory factor analysis. Missing data will be handled by multiple imputation and full information maximum likelihood.

### 2.9. Data protection

The questionnaires will be administered electronically and stored in Services for Sensitive Data (TSD), (https://www.uio.no/english/services/it/research/sensitive-data/about/index.html). Names of participants will be stored in separate files, only accessible by the project leader and data administrator. Deidentified data will be transferred to national and international collaborators through TSD. The teachers and the parents are informed about this process in their informed consent forms.

### 2.10. Participant remuneration

Schools participating in the study will not be compensated for the time used to implement the intervention and participate in the RCT. However, after the study has ended, the principal or school contact person will receive a small gift as an appreciation for their work and effort.

### 2.11. User involvement

During the development of the intervention, three focus group interviews involving 18 pupils were conducted with pupils from two secondary schools, to gain knowledge about their experiences with sexual and gendered harassment in school and how school personnel respond to harassment. (These schools were not a part of the current study). Two of the interviewed pupils have further contributed with feedback to the development of the pilot intervention that was tested in 2018 in order to assess pupils' and teachers' experiences with the intervention. One of these pupils and another youth have also contributed with feedback about the modification of the intervention which was later named “Stop Sexual Harassment” as well as the planning of the RCT study.

## 3. Method and analysis: Process evaluation

### 3.1. Study design

In this study, a process evaluation with focus group interviews will also be conducted. The study will thus utilise qualitative data to assess implementation quality and intervention fidelity.

### 3.2. Recruitment of participants

All principals in the intervention group were invited by e-mail or phone to participate in a process evaluation of the intervention. Three schools from wave 1 volunteered for focus group interviews. However, the interviews were cancelled due to national COVID-19 imposed regulations restricting pupils to meet between classes and a high number of pupils and teachers being absent from school. Online meetings were not considered, as participation would require individual rooms for participants at each class. It was decided to postpone the focus group interviews to wave 2. For wave 2, three schools will be invited to participate in the focus group interviews. At each school, there will be one interview with six pupils, and one interview with six teachers. The recruitment of teachers and pupils to focus group interviews will be conducted by the principals, with the instruction to balance the informant group with regard to gender and age. Prior to participation in the interviews, parents, pupils and teachers will be given information about the process study and asked to sign consent/assent forms.

### 3.3. Data collection procedure

Qualitative data on implementation quality, intervention fidelity, and engagement with the intervention will be collected through separate focus group interviews with pupils and teachers who have participated in the intervention. Pupils and teachers will be interviewed at their respective schools during school hours. Each interview will last about 90 min and will be audiotaped. Survey data (see description below) will also be used to assess intervention fidelity.

### 3.4. Process measures; Implementation quality and fidelity

Overall implementation quality, intervention fidelity, and engagement with the intervention will be assessed by conducting focus group interviews with pupils and teachers.

The focus group interview with teachers will ask about their experiences with delivering each of the eight lessons, and the intervention as a whole. In particular, the teachers will be asked what they liked and did not like about the lectures, and why. If the teachers skipped a lesson, they will be asked why this particular lesson was not delivered.

The focus group interview with pupils will ask about their experiences with each of the eight lessons and the intervention as a whole. In particular, the pupils will be asked what they liked and did not like about the classes, and why.

Adherence to the protocol will be assessed with questions answered by both teacher and pupil questionnaires at T2 as a part of the RCT (see section 2: Method and analysis: Randomised controlled trial). Here the teachers will be asked to what extent they completed the digital training for teachers, and to what extent they delivered the lessons and class activities. The pupils will be asked about their participation in the lessons and milestone activities.

### 3.5. Data analysis

The audiotaped interviews will be transcribed and uploaded to an electronic software for qualitative data analysis (NVivo). The interviews will be analysed by the use of Systematic text condensation (54), which involves a structured process to assess the strength and weakness of the content of and implementation of the intervention.

### 3.6. Data protection

The focus group interviews will be administered by offline recorders and digital recorders by the use of Nettskjema, and stored at TSD. Only the project leader and the person transcribing the interviews will have access to the audio interviews, which will be deleted once they are transcribed. Transcribed and deidentified interviews will be transferred to collaborators through TSD. The teachers and the pupils' parents are informed about this in their respective consent forms.

### 3.7. Participant remuneration

Pupils and teachers who participate in the focus group interviews will each receive a gift certificate for a movie theatre ticket.

## 4. Discussion

This paper describes a Randomised Controlled Trial (RCT) evaluating the effect and implementation of a new universal prevention school-program “Stop Sexual Harassment” in Norway. Outcomes assessed are reductions in sexual and gendered harassment among pupils, and increases in teachers' detection of and responses to the harassment. The intervention will be administered during school hours in Norwegian secondary schools.

A strength of the intervention “Stop Sexual Harassment” is that the program is both theory-driven (32, 33) and grounded in empirical research (34, 36–38, 40). Another strength is the flexibility of the program, in that it takes into consideration that the degree and manifestation of sexual and gendered harassment may vary among schools. Each school will address how harassment is identified as a problem in their school, and tailor how they use the program accordingly. If harassment is not observed in an individual school, the program may still be useful for preventative purposes.

A weakness of the study is that the recruitment of schools has been challenging, and the school withdrawal rate was high. Even though this may primarily be attributed to the COVID-19 pandemic and its restriction on schools, the external validity of the intervention may be questioned.

Another weakness of the study is that several of the questionnaire instrument have been modified. Thus, the validity and reliability of these instruments are uncertain.

In addition to evaluating the intervention's effectiveness, the study includes a process evaluation that may explain *why* the intervention may have had or failed to have an effect. The process evaluation aims to discover facilitators and barriers to a successful implementation of the intervention. With this knowledge, we can revise the program by improving the focus, content and implementation strategies. Assuming that the program has positive effects, large-scale implementation of the program may be offered for secondary schools. The educational material for teachers will be made publicly available providing that schools agree to complete the full program. This to ensure that teachers and school administration react to the harassment (second part of the program), once this has been identified and reported by the pupils (first part of the program).

## Ethics statement

The studies involving human participants were reviewed and approved by the Regional Committees for Medical and Health Research Ethics, South-East (Norway) (REK: 231585), and Norwegian Centre for Research Data (NSD). Written informed consent to participate in the study will be obtained from the participants legal guardian.

## Author contributions

HS designed the intervention, acquired funding for the project, and drafted the manuscript. KB supervised HS and commented on the manuscript regarding the randomised controlled trial. BH, RB, and VP commented on the manuscript involving the RCT. TF and TT commented on the manuscript involving the process evaluation. All authors read and agreed to be accountable for the content of the manuscript.
